# Differential Gene Expression and Protein Abundance Evince Ontogenetic Bias toward Castes in a Primitively Eusocial Wasp

**DOI:** 10.1371/journal.pone.0010674

**Published:** 2010-05-17

**Authors:** James H. Hunt, Florian Wolschin, Michael T. Henshaw, Thomas C. Newman, Amy L. Toth, Gro V. Amdam

**Affiliations:** 1 Departments of Biology and Entomology and W. M. Keck Center for Behavioral Biology, North Carolina State University, Raleigh, North Carolina, United States of America; 2 National Evolutionary Synthesis Center, Durham, North Carolina, United States of America; 3 Department of Chemistry, Biotechnology and Food Sciences, Norwegian University of Life Sciences, Ås, Norway; 4 School of Life Sciences, Arizona State University, Tempe, Arizona, United States of America; 5 Department of Biology, Grand Valley State University, Allendale, Michigan, United States of America; 6 Department of Entomology, University of Illinois at Urbana-Champaign, Urbana, Illinois, United States of America; 7 Department of Entomology, Pennsylvania State University, University Park, Pennsylvania, United States of America; University of Bristol, United Kingdom

## Abstract

*Polistes* paper wasps are models for understanding conditions that may have characterized the origin of worker and queen castes and, therefore, the origin of paper wasp sociality. *Polistes* is “primitively eusocial” by virtue of having context-dependent caste determination and no morphological differences between castes. Even so, *Polistes* colonies have a temporal pattern in which most female larvae reared by the foundress become workers, and most reared by workers become future-reproductive gynes. This pattern is hypothesized to reflect development onto two pathways, which may utilize mechanisms that regulate diapause in other insects. Using expressed sequence tags (ESTs) for *Polistes metricus* we selected candidate genes differentially expressed in other insects in three categories: 1) diapause vs. non-diapause phenotypes and/or worker vs. queen differentiation, 2) behavioral subcastes of worker honey bees, and 3) no *a priori* expectation of a role in worker/gyne development. We also used a non-targeted proteomics screen to test for peptide/protein abundance differences that could reflect larval developmental divergence. We found that foundress-reared larvae (putative worker-destined) and worker-reared larvae (putative gyne-destined) differed in quantitative expression of sixteen genes, twelve of which were associated with caste and/or diapause in other insects, and they also differed in abundance of nine peptides/proteins. Some differentially-expressed genes are involved in diapause regulation in other insects, and other differentially-expressed genes and proteins are involved in the insulin signaling pathway, nutrient metabolism, and caste determination in highly social bees. Differential expression of a gene and a peptide encoding hexameric storage proteins is especially noteworthy. Although not conclusive, our results support hypotheses of 1) larval developmental pathway divergence that can lead to caste bias in adults and 2) nutritional differences as the foundation of the pathway divergence. Finally, the differential expression in *Polistes* larvae of genes and proteins also differentially expressed during queen vs. worker caste development in honey bees may indicate that regulatory mechanisms of caste outcomes share similarities between primitively eusocial and advanced eusocial Hymenoptera.

## Introduction

Determination of morphologically and behaviorally distinct worker and reproductive castes occurs during larval development in ants [1], honey bees and stingless bees [2], [3], vespine wasps [4], [5], and many (but not all) swarm-founding paper wasps [6], all of which represent “advanced eusocial” [7] insects. In contrast to advanced eusocial insects, worker and reproductive castes in “primitively eusocial” [7] insects are not morphologically distinct [3]. For example, in most species of paper wasps (*Polistes*) both workers and reproductives encompass the full range of body sizes [8], [9]. Therefore, a question that bears directly on conditions presumed to have characterized the threshold of insect social evolution [10] is to ask whether the differentiation of workers and gynes (presumptive foundresses and, therefore, presumptive future queens) in anatomically monomorphic “primitively eusocial” Hymenoptera also has an ontogenetic component, as in advanced eusocial forms.

Primitively eusocial paper wasps of the genus *Polistes* have long been used as models to investigate behavioral aspects of sociality in the wasp family Vespidae [11]. *Polistes*' worker/queen caste determination occurs in the adult stage [12] and may be determined by constraints on breeding [13] such as physiological costs of work undertaken by workers [14] and/or behavioral dominance of workers by queens [15]. Worker caste determination is not random, however, as *Polistes* colonies are characterized by a temporal pattern in which workers precede non-workers of the same generation [9], [16], [17], although both early non-workers [18] and late workers [19], [20] may occur at low frequencies [9], [21]. Hunt and Amdam [21] recognized that any adult female *Polistes* can become either a worker or queen and that worker/queen castes are determined among adults, but they proposed a specific hypothesis for a process whereby larval developmental divergence yields females reared early in the colony cycle that are biased to become workers and females reared later in the colony cycle that are biased to become gynes.

Multiple adult features have led previous investigators to suggest that the behavioral castes of primitively eusocial wasps are preceded by developmental divergence during larval growth [10]. The evidence includes apparent morphological caste dimorphism in *Polistes olivaceous* [22], [23] and *Belonogaster petiolata* [24], unequivocal morphological caste dimorphism in *Ropalidia galimatia* [25], and two phenotypes of reproductive development in newly-emerged *Ropalidia marginata* [26], [27], [28]. Abdominal fat in adult female *Polistes* reared early in the colony cycle is scant and yellow, whereas abdominal fat in females reared late in the colony cycle is abundant and white [29], [30], [31], [32]. Although ovarian development is slight in both workers and gynes, it is significantly greater in workers [32]. If these adult morphological and physiological differences reflect a divergence during larval development that has the potential to bias larvae toward either worker or gyne caste, we reasoned that gene expression differences during larval development should exist. An expressed sequence tag (EST) project based on 454/Roche pyrosequencing, which yielded partial sequences for ca. 3,000 genes for *Polistes metricus* [33], can be a resource to test the hypothesis that *Polistes* females diverge during larval life onto two physiological tracks. In the first utilization of the EST resource, quantification of twenty-eight gene transcripts in the mature adult brain revealed that workers differed significantly from gynes that were the workers' same-generation siblings [33], a result corroborated in a large scale study that focused on brains of the same groups and included transcript profiling, via microarray, of approximately 3000 genes [34]. These results are consistent with the hypothesis that *Polistes* workers and gynes are physiologically different despite being anatomically indistinguishable. The differences documented in [33], [34] could reflect differential outcomes of early developmental events [21], [35]. However, the data are based on adult sample material rather than larvae, thus they do not directly test the developmental divergence hypothesis.

Using the *P. metricus* EST resource, we designed research on larvae that would bear directly on developmental events during the larval stage. We predicted that if divergent developmental physiology underlies the difference between workers and gynes, then levels of gene and protein products should differ between worker-destined and gyne-destined larvae. We tested the predicted divergence by comparing field-collected foundress-reared (putative worker-destined) and worker-reared (putative gyne-destined) fifth (final) instar larvae of *P. metricus* in two ways. One study focused on the mRNA levels of 38 specifically-targeted candidate genes, and another study used a non-targeted proteomics approach [36].The mRNA candidate gene study was based on *a priori* expectations of difference. The proteomics analysis was non-targeted to enable an unbiased evaluation of foundress-raised vs. worker-raised larvae without *a priori* expectations. Thus, the proteomics analysis was an independent test that could identify new physiological markers as well as confirm or dismiss mRNA expression differences, thereby enabling added confidence in our data evaluation. Both methodological approaches have the potential to reveal the occurrence and characteristics of molecular separation between the two types of larvae.

## Methods

### Field Collections

Our study taxon, *Polistes metricus* is a common paper wasp in the Midwestern and Southeastern US. Specimens were collected in spring 2005 and 2007 from nests of *P. metricus* naturally-founded by solitary foundresses in “nest boxes” at two field sites near St. Louis, Missouri, USA, as previously described [37]. Nests were not used in other studies, and they were undisturbed after marking the foundress with a dot of paint until the dates of specimen collections. Nests were selected for larval collections only if all nest cells contained apparently normal brood. At collection, forceps were used to remove larvae from their nest cells. Each larva was flash frozen within seconds in liquid N_2_, transferred to a labeled tube on dry ice, and stored at −80°C. One set of specimens was collected in mid-June, when the single foundress was the only adult wasp present. A second set was collected in the same manner from other nests in late July and early August, when the foundress was accompanied by a minimum of three workers. Specimens for this study were restricted to large fifth-instar larvae that were of a size that soon would begin spinning their pupal cocoon. Specimen collection data and causes for excluding 7 specimens are presented in Supplementary Table S1.

### Sex Determination

Only females become workers or gynes, therefore male larvae must be excluded from analysis. In Hymenoptera, females are diploid while males are haploid [38]. Accordingly, we used a tissue chip from the posterior of some 2005 foundress-reared larvae and all worker-reared larvae to genotype 6 microsatellite loci. Female larvae were heterozygous at two or more loci and thus diploid. This approach reliably excludes haploid males. In certain circumstances, diploid eggs can develop into males [39], and this approach could fail to exclude such males. However, the production of such diploid males would be marked by the production of early males along with the first cohort of workers, and no such males were detected, indicating that diploid male production did not occur on any of the study nests. Methods and loci are described in [37].

### Gene Selection and Primer Design

Thirty-eight genes were chosen for mRNA expression via real time quantitative reverse transcription polymerase chain reaction (qRTPCR). Microarray analysis, now available for *P. metricus* [40] was not an option for this study; at the time of analysis (2006–2007) the resource was not developed, and cost constraints left us unable to print our own custom-made arrays.

Because diapause physiology may underlie developmental divergence in *Polistes* [21], literature was searched for genes differentially expressed in non-diapause vs. diapause phenotypes in other insect taxa (Table 1). Additional candidate genes were known to be differentially expressed in worker-destined vs. queen-destined honey bees (*Apis mellifera*), bumble bees (*Bombus*), and a stingless bee (*Melipona quadrifasciata*) (Table 1). We previously found a hexameric storage protein to be differentially expressed in putative worker-destined vs. gyne-destined larvae of *P. metricus* [37], [41], therefore it is particularly meaningful that one candidate gene encodes hexameric storage proteins. The absence/presence of hexameric storage protein could be a key physiological mediator of diapause-related larval pathway divergence, perhaps as a regulator of juvenile hormone (JH) in foundress-reared (low hexamerin, high JH) vs. worker-reared (high hexamerin, low JH) newly-emerged adults [21]. We also quantified expression of an endogenous control gene, *RSP8*, as used in studies of *A. mellifera* [42], [43], [44].

**Table 1:** *Polistes metricus* putative genes quantified in this study, their full name and/or putative function, and the contig/read number.

*P. metricus* putative gene	Full name and/or putative function	contig/read	D	Q/W	*A.m.* behavior
*PmAcCoAS*	Acetyl coA synthetase	Contig41318			
*PmCG11971-like*	CG11971-like, nucleic acid and Zn binding	Contig21820			[45]
*PmCG5237-like*	PmCG5237-like, Zn ion binding	Contig44641			
*PmCG9005-like*	PmCG9005-like, no known function in *Drosophila*	Contig4643			[45]
*Pm chymotrypsin precursor*	chymotrypsin precursor	Contig33054		[72]	
*PmClock*	Clock, circadian activity	Contig27898			
***PmeELF-1a***	Translation elongation factor 1a	Contig44948			
*PmEfl21*	Essential for life 21, embryonic development	Contig46745			
*Pmendopep*	Endopeptidase inhibitor	Contig32095			[45]
*PmFAS*	Fatty acid synthase	Contig43529		[83]	
*Pmfax*	Failed axon connections, axonogenesis	Contig42680			[45]
*Pmg5sd*	Glutathione 5 S transferase	Contig34111			[45]
***PmHex70b (a)***	Hexamerin 70b	Contig40833	[**68**]	[**46**]	
***PmHex70b (b)***	Hexamerin 70b	Contig45913	[**68**]	[**46]**, [72], [73****]	
*PmHsp1alpha*	Heat shock protein 1 alpha	Contig46714	[63], [66]		
***PmHSC70***	Heat shock cognate 70	Contig46725	[**65**]	[**84**]	
*PmHsp90*	Heat shock protein 90	Contig45687	[63]		
***PmHSP90alpha***	Heat shock protein 90 alpha	Contig44208	[**63**]		
***PmILP2***	Insulin-like peptide 2	Contig16852		[**47**]	[**44**]
*PmInos*	Inositol 3 phosphate synthase	285736 3178 0687			[45]
*PmInR1*	insulin receptor 1	203959 4018 1073		[48]	[44]
*PmInR2*	insulin receptor 2	Contig16872		[48]	[44]
***PmIRS***	insulin receptor substrate	Contig18215		[**48**]	
*Pmmcp*	monocarboxylate porter, carbohydrate transport	168197 3721 2905			[45]
***Pmoxidoreductase***	CG6910-like oxidoreductase	Contig39053		[**46**]	[**45**]
*PmPCNA*	proliferating cell nuclear antigen	Contig39238	[62], [85]		
***PmPi3K***	phosphatidyl inositol 3 kinase	Contig15991	[**69**]		[**45**]
***PmRfaBp***	retetinoid/fatty acid binding protein	Contig46778		[**84**]	[**45**]
***PmAPO***	apolipoprotein	Contig46673		[**73**]	
***PmSh3Beta***	Sh3Beta, no known function in *Drosophila*	Contig40061			[**45**]
***PmSPARC***	SPARC, cell adhesion, mesoderm development	Contig15503			[**45**]
*PmTctp*	translationally controlled tumor protein	Contig45632			[45]
***Pmtif2B***	translation initiation factor 2B	Contig26018			[**45**]
*PmTor*	target of rapamycin, growth and response to nutrients	Contig27724		[49]	
***PmTPX1***	thioredoxin peroxidase 1	Contig46225		[**72**]	
***PmTPX3***	thioredoxin peroxidase 3	Contig43747		[**72**]	
*Pmtungus*	tungus, Memory formation	Contig45573			[45]
***Pmusp***	ultraspiriracle, juvenile hormone binding	Contig37120	[**68**]		
*PmVg1*	vitellogenin 1, egg yolk protein	Contig46807		[73]	[59]

**Figure 1:**
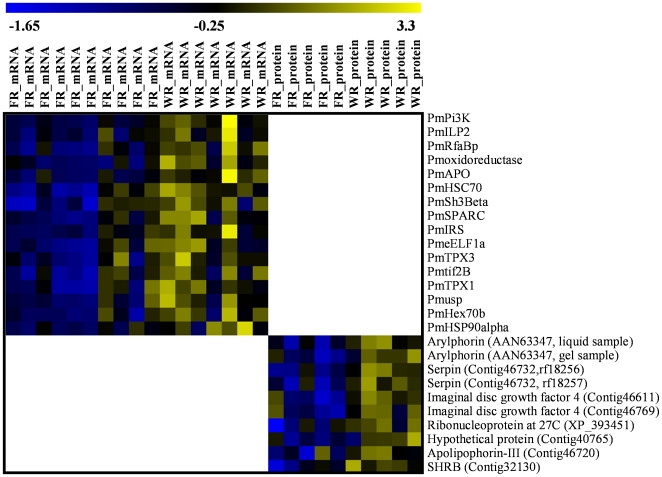
Heat map visualization of relative expression of sixteen genes and nine peptides. Sixteen genes (upper) and 9 peptides (lower) significantly differentially expressed in foundress-reared (FR; putative worker-destined) vs. worker-reared (WR; putative gyne-destined) *P. metricus* fifth-instar larvae. Bar at top gives color scale of relative expression from the mean expression: downregulation  =  blue; upregulation  =  yellow. Z-transformed transcript and peptide/protein abundances visualized using TMeV version 4.3.02.

*Polistes metricus* putative genes quantified in this study together with their full name and/or putative function and the contig/read number for each gene based on the assembly of data from the *P. metricus* EST data set as presented in [33]. Columns ‘D’ and ‘Q/W’ give reference sources. D  =  diapause-related in various insects, Q/W  =  queen/worker caste-related in highly eusocial Hymenoptera. Column ‘*A.m.* behavior’ gives literature sources for genes known to be expressed differently in correlation with adult behaviors of nest workers vs. foragers of the honey bee *Apis mellifera*. Genes without literature citations were non-candidate genes for which primers were readily available. Cases with references for a single gene in either/or columns ‘D’ and ‘QW’ that are also the ‘*A.m.* behavior’ column are described in the discussion. Boldface type identifies the sixteen genes significantly differently expressed between foundress-reared (putative worker-destined) and worker-reared (putative gyne-destined) larvae (see also Figure 1) and the categories in which those significant differences occur.

Differences might occur between the two sets of collected larvae that would reflect differences in rearing conditions such as day length, temperature, or food resource availability rather than a developmental bias that would be revealed by the candidate genes. To begin to address this possibility, we selected a set of non-candidate genes related to foraging and differentially expressed between two adult behavioral castes of worker honey bees, nurses that perform tasks within the hive and foragers that perform tasks in the field [45]. These genes are not among those found to be differentially expressed between developing larvae that become either worker or queen honey bees [46], [47], [48], [49]. Several genes for which we had no *a priori* expectation of an association with caste development or foraging were included in the study as non-candidate genes based on ready availability of primers for them. One of these non-candidate genes, *apolipoprotein*, was *a priori* suspected of possible involvement in larval divergence because of the known difference of two types of abdominal fat in worker and gyne *Polistes*, but in the absence of documentation that it plays a role in either of our candidate gene categories, it was placed conservatively as a non-candidate gene.

Published sequences for candidate and non-candidate genes were used to search the *Polistes metricus* EST transcriptome (http://stan.cropsci.uiuc.edu/454/blast/waspblast.html) for BLAST sequence matches. If a match was found, primers were designed using Primer Express Version 2 (Applied Biosystems) using the DNA PCR module for qPCR using SYBR Green. Prior to design, sequences were screened for possible indels in A and T homopolymer runs. The sequences were translated, and BLASTP [50] was used to compare sequences to honey bee proteins to look for correctable frame shifts. Primer sequences are given in Table S2.

### Gene Transcript Quantification

For the 2005 collections, total RNA was extracted from ca. 80% to 90% of each foundress-reared larva that remained after tissue chip removal (Sex Determination, above) or from whole foundress-reared and worker-reared larvae. Tissues were homogenized in Trizol reagent (Invitrogen) with a motorized pestle and centrifuged at 4°C. The aqueous phase was mixed with an equal volume of 70% EtOH and transferred to a Qiagen RNeasy mini spin column followed by the RNeasy extraction protocol. RNA quality was examined using an Agilent BioAnalyzer and its concentration quantified with a Nanodrop spectrophotometer. Two hundred ng of RNA were used for cDNA synthesis with ArrayScript, and quantification was performed as in [33]. For each sample, quantitative PCR was performed in triplicate using 1.5 ng cDNA in 10 µl reactions composed of 2X-SybrGreen Master Mix (Applied Biosystems) and a final concentration of 0.25 µM gene-specific primers. 386-well plates were run on the Applied Biosystems 7900 HT System Detection System and analyzed with SDS version 2.2 software. Absolute quantification was determined using a genomic DNA standard curve.

### Protein Quantification

The 2007 specimens were extracted using Trizol reagent as in 2005. Proteins were precipitated out of the phenol-chloroform phase (200 µg per sample) using a methanol/chloroform protocol [51] and redissolved in 40 µl Tris pH 8.5, 6 M urea, 2 M thiourea, 5 mM CaCl_2_, and 0.15 M NaCl. Samples then were vortexed and diluted in 160 µl Tris pH 8.5, 5 mM CaCl_2_, and 0.15 M NaCl. Protein concentration was determined by the Bradford assay. Samples were split, and 40 µg of each sample were subjected to SDS-PAGE followed by tryptic digestion or to direct in-liquid digestion as described previously [52].

SDS-PAGE was performed at 120 mV for 20 min. Each band was divided into five major pieces, and proteins were digested according to the following procedure: each gel piece was washed twice with 200 µl of NH_4_HCO_3_ followed by three washes with 50% acetonitrile/50% NH_4_HCO_3_. Gel pieces were then dried in a speed vac at 30°C for 15 min, followed by the addition of 10 µl of a 5 µg/ml protein standard (beta-lactoglobulin C7880-10MG in 0.15 M NaCl). Samples sat on ice for 10 min and were then dried in a speed vac at 30°C for 10 min. Subsequently, 20 µl trypsin solution (12.5 ng of trypsin/µl digestion buffer [25 mM NH_4_HCO_3_, 10% acetonitrile, 5 mM CaCl_2_]) were added, and the samples were incubated at 4°C for 30 min followed by the addition of 60 µl of digestion buffer. Proteins were digested overnight at 30°C, and peptides were extracted as described in the manual for Roche sequencing grade trypsin.

### LC-MS/MS Analysis

Peptides were analyzed by LC-MS/MS as described previously [52]. Briefly, about 20 µg of in-liquid digested sample or the entire result from one in-gel digest were subjected to HPLC, and peptides were eluted directly into a linear ion trap (LTQ, Thermo Electron, San Diego, USA). Peptides were separated on a monolithic column (100 µm ID, Merck, Darmstadt, Germany) using a 105 min gradient ranging from 95% A (0.1% formic acid, 99.9% H_2_O) to 80% B (0.1% formic acid, 99.9% acetonitrile) for in-liquid digested samples and a 65 min gradient for in-gel digested samples, respectively. The top five most abundant MS peaks were automatically selected for MS/MS. OMSSA 2.0.0 [53] was used to run the experimentally obtained spectra against a database containing the information of the *P. metricus* transcriptome contigs translated into all 6 possible reading frames as well as keratin, trypsin, and beta-lactoglobulin sequences. Only fully tryptic peptides were considered, and a maximum of two missed cleavages was accepted. Deamidation of N and Q, and methione oxidation were allowed as variable modifications. MS and MS/MS error tolerance was set at 0.8 Da.

### Data Analysis

mRNA expression data were analyzed using R statistical software. For three of the 38 genes (*PmSh3β*, *PmInR2*, and *PmHSP90α*), the same samples were run two times, so the data were average for the two replicates of these genes. In addition, two different contigs, *PmHex70b (a)* and *PmHex70b (b)* of Table 1, were found to correspond to the same *P. metricus* gene, thus their mRNA expression data, which were highly correlated (Pearson R = 0.98, p<0.0001), were averaged for analysis. All data were log transformed to normalize the residuals and divided by the expression values for each individual of *PmRSP8* (NCBI accession #TI1888782351), which showed minimal variation across groups and individuals, as an endogenous control. Each gene was tested for differences between foundress-reared and worker-reared larvae using both ANOVA (lme function in R) and a non-parametric Mann-Whitney U test (wilcox.test function in R). Missing values (2 in total) were replaced with the median of the sample group for the respective gene before subjecting the data to the Mann-Whitney U test. In order to adjust for false discovery rate caused by repeated Mann-Whitney U testing, we used a type I error bootstrap correction with 1000 iterations per generation following an established procedure for large scale datasets coded by Matlab R2007b [36], [52], [54], [55]. This approach established a corrected p-value cutoff at p≥0.1. Linear discriminant analysis (LDA, using the lda function in R) was performed on 16 genes that were significantly different based on both the ANOVA and Mann-Whitney U approaches (see *Results*). However, because the LDA failed due to high colinearity, we performed Pearson correlations (cor function with “Pearson” option in R) to identify which genes had highly correlated gene expression. The analysis identified several highly correlated genes that can be closely functionally related. *PmPi3K* was correlated with two other insulin pathway genes, *PmILP2* (0.93) and *PmIRS* (0.954). *PmILP2* was also correlated with *PmIRS* (0.948), *PmRfaBp* was correlated with two other lipoprotein genes, *PmAPO* (0.912) and *PmHex70b* (0.916), and *PmeELF1a* was correlated with *PmSPARC* (0.939). To eliminate the colinearity problem of LDA we removed *PmPi3K*, *PmILP2*, *PmRfaBp* and *PmeELF1a* before repeating the LDA analysis on the remaining 12 genes. The resulting linear discriminant function was used as a basis for leave-one-out cross validation (using the lda function in R with the “CV” option) to confirm the ability of the gene expression differences to correctly classify individuals as foundress-reared or worker-reared. Test scores and associated p values for both the Mann-Whitney U test and ANOVA are given in Table S3.

Peptide quantities were analyzed using Statistica 6.0 (Mann Whitney U-tests) and Matlab R2007b (bootstrap correction for type 1 error, as for gene expression data above). Statistical analysis was based on spectral count [52], however the normalization procedure was specific to in-gel and in-liquid digested samples. For the in-gel digested samples, spectral counts were first normalized to the individual values for beta-lactoglobulin to account for possible differences in the digestion procedure. This was followed by normalization to the total spectral count for all bands of one lane to account for possible loading differences. Liquid samples were normalized to total spectral count only. Using a decoy database, the false discovery rate was determined to be ≤1%. Correction for type 1 error resulted in a corrected p-value cut-off equivalent of p≤0.05. Only hits with a spectral count of ≥3 in at least 3 of 5 replicates were considered.

## Results

### Sex Determination

Microsatellite analysis of 13 foundress-reared specimens from 2005 showed all to be females. A specimen for which no microsatellites amplified was eliminated from further analysis (Table S4a). All other foundress-reared specimens were assumed to be females, as has consistently been the case in studies at the same study sites [37], [56], [57]. Eighteen of 29 worker-reared larvae were males, and one specimen was triploid (Table S4a). Sample sizes for gene expression analyses were 10 foundress-reared (putative worker-destined) and 7 worker-reared (putative gyne-destined). In 2007 only 6 of 23 worker-reared specimens were females (Table S4b). Sample sizes for peptide quantifications were 5 foundress-reared and 5 worker-reared (Table S1).

### mRNA Expression

**Figure 2:**
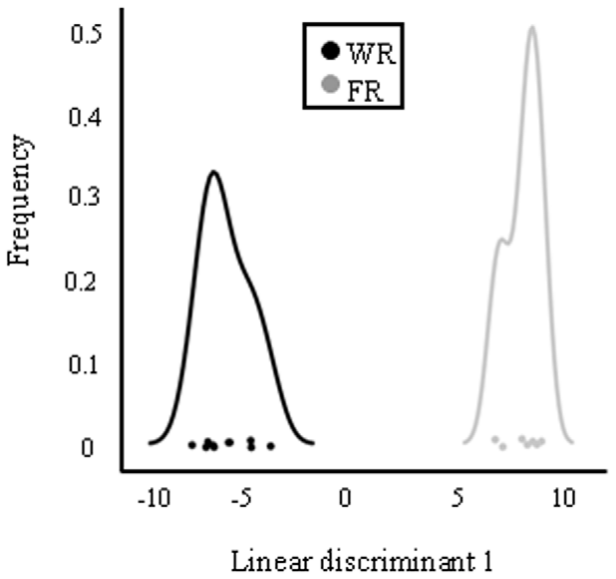
Linear discriminant (LD) analysis of 12 genes significantly different at the mRNA level. Linear discriminant analysis of 12 genes significantly different at the mRNA level clearly separated foundress-reared (FR; putative worker-destined) and worker-reared (WR; putative gyne-destined) larvae. X axis  =  LD value; Y axis  =  LD value frequencies. 88% of individuals were correctly classified as FR or WR based on LD values.

We quantified the expression of 38 genes (Table 1). Both the corrected Mann-Whitney U test and mixed model ANOVA identified the same 16 genes to be significantly differentially expressed between the two sets of larvae (Figure 1; Table 1). All 16 significantly differentially expressed genes were up-regulated in worker-reared (gyne-destined) larvae (Figure 1). Linear discriminant analysis of 12 significant genes succeeded in clearly separating the two groups from each other (Figure 2). For explanation of the 12 genes used for this analysis, see Methods: *Statistical Analyses*. Leave-one-out cross-validation classified 15 out of 17 (88%) as foundress- or worker-reared.

### Protein Quantification

**Figure 3:**
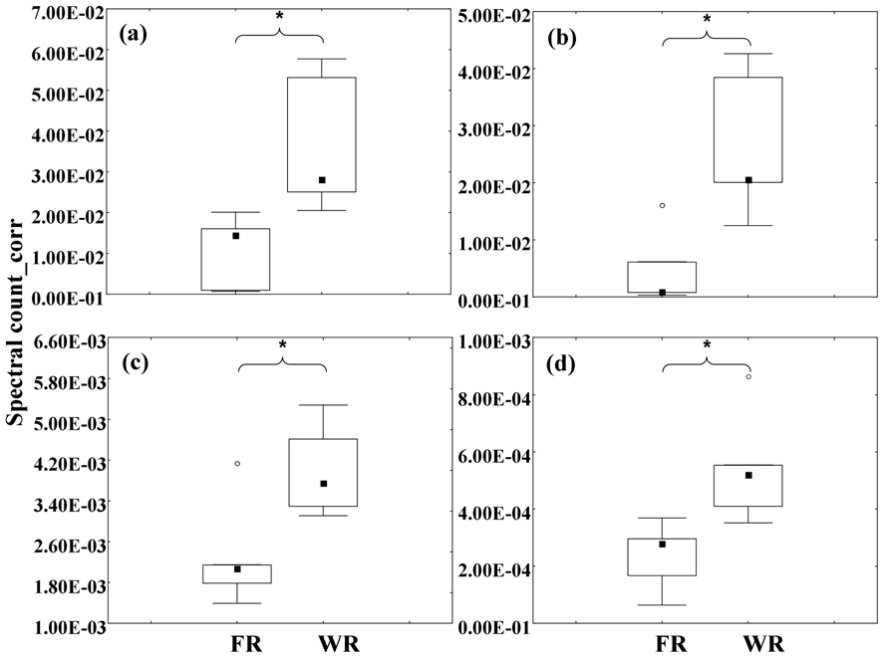
Quantities of four peptides/proteins significantly differentially expressed in foundress-reared vs. worker-reared *P. metricus* fifth-instar larvae. Quantities of four peptides/proteins significantly differentially expressed in foundress-reared (FR; putative worker-destined) vs. worker-reared (WR; putative gyne-destined) *P. metricus* fifth-instar larvae: median (dark squares), 25^th^-75^ th^ percentiles (brackets), and outliers (open circles). Stars denote significant differences (Mann Whitney U-test, p≤0.05, n = 5 per group). Y-axis: spectral count for individual peptides/proteins corrected for overall spectral count. Sequences are homologous to: (a) arylphorin of the boll weevil (NCBI accession AAN63347); (b) serpin (contig 46732, rf 18257); (c) imaginal disc growth factor 4 (contig 46769); (d) shrub (contig 32130).

Quantification of expression was performed on putative proteins, as identified by database entry hits. One or more peptides of a single mature protein can belong to one or several database entry hits, thus one protein might produce more than one hit entry. Of these database entry hits, 9 were significantly quantitatively different between the two sets of larvae (Figure 1; Table S5), and 7 were supported by at least two distinct peptides (Table S5). The hits included a peptide homologous to the hexameric storage protein arylphorin from the boll weevil, *Anthonomus grandis*, and that protein was identified in both in-gel and in-liquid digestion samples. Other hits indicated that several peptides homologous to an imaginal disc growth factor were up-regulated in gyne-destined larvae: a nuclear ribonucleoprotein; shrub; serpin; and apolipophorin-III (Figure 1; Table S5). Figure 3 illustrates the magnitude of differences for arylphorin, serpin, imaginal disc growth factor 4, and a shrub-like protein.

## Discussion

By targeted testing of 38 genes and non-restricted screening of protein levels, we found that mRNA expression of 16 of 38 quantified genes and quantities of 9 peptides/proteins were significantly up-regulated in worker-reared (putative gyne-destined) vs. foundress-reared (putative worker-destined) fifth instar larvae of *Polistes metricus*. The gene expression differences represent transcriptional differences at the time of specimen collection, while the differentially expressed peptides/proteins represent readily available stored protein of high abundance rather than active differences in transcription. Thus, our finding that none of the differently-expressed genes are represented among the differently-expressed peptides/proteins is consistent with processes commonly observed in molecular biology: transcript abundance does not always translate into protein abundance, and turnover rates can differ between mRNA and protein [58].

Because the larval developmental divergence that we hypothesize could incorporate genes related to diapause and to caste differences in highly eusocial insects, we had searched literature to find candidate genes in those two categories. Two candidate genes, *oxidoreductase* and *Vg1*, belonged in both categories. Non-candidate genes had been drawn from loci that are differentially expressed between behaviorally different nurse and forager honey bees. A few additional genes for which primers were readily available were included in the non-candidate gene category. In publications subsequent to our gene expression quantification, two non-candidate genes were documented to be in one of our candidate categories: *Pi3K* is diapause-related, and *RfaBp* plays a role in queen/worker differentiation [59]. Correspondingly, three of our candidate genes, *ILP2*, *InR1*, and *InR2*, were found to be implicated in honey bee nurse/forager regulation [44]. In addition, *apolipoprotein*, which had been classified as non-candidate due to the absence of documentation of its possible involvement in diapause or caste relatedness, was shown to play a role in queen/worker larval divergence. These overlaps in our classification of genes as candidate or non-candidate are not altogether surprising, given the pleiotropic nature of many genes as well as the suggestion that behavior differences among worker honey bees reflect in part an underlying ground plan of reproductive physiology [60], [61]. These considerations make it biologically difficult to draw a clean contrast between our candidate genes and those non-candidate genes that came from studies of worker honeybees, In consequence, we cannot definitively exclude the possibility that our data reflect, at least in part, generalized expression differences that include genes both implicated and not implicated in pathways that could regulate larval developmental divergence. Nonetheless, one particular result provides support for specific rather than generalized gene expression differences: 12 of 20 (60%) candidate genes are differentially-expressed, whereas 4 of 18 (22%) non-candidate genes are differentially expressed.

The gene encoding heat shock protein 90α is up-regulated, and the protein itself is down-regulated, during pupal diapause of the flesh fly, *Sarcophaga crassipalpis* [62], [63]. We found a gene encoding this protein to be up-regulated in worker-reared larvae of *P. metricus*. Heat shock proteins are widely implicated as playing roles in insect diapause [54], [63], [64], [65], [66], as are hexamerins [54], [67], [68] and several genes in the insulin pathway [69], [70], [71]. Hexameric storage proteins are differentially regulated between queen-destined vs. worker-destined larvae of *A. mellifera* [46] and *B. terrestris* [72]. We found up-regulation of *PmHex70b*, *PmHSC70*, and arylphorin protein in gyne-destined larvae. Hexamerin 2 was up-regulated in adult queens vs. workers of the tropical paper wasp *P. canadensis* [73], suggesting that the possible interplay of hexamerin expression and caste bias is not limited to *Polistes* species with exclusively temperate zone distributions. Note that the evolutionary relationships among the mRNA sequence for *PmHex70b* and the unsequenced gel bands previously called hexamerin 1 and 2 of [37] and hexamerin 2 of [73] remain to be ascertained.

Transcripts of three genes that are part of insulin/insulin-like signaling pathways and play roles in growth, reproduction, and aging (*PmPi3K*, *PmILP2*, and *PmIRS*) are up-regulated in worker-reared (putative gyne-destined) *P. metricus* larvae, which emerge from pupation in apparent reproductive diapause [35], [37]. In possible contrast to our finding, down-regulation of insulin signaling can set the stage for ovarian diapause in *Drosophila* [69] and *Culex pipiens* [71]. Differences in life history stage at which diapause occurs could explain inter-taxon differences in diapause signatures that incorporate analogous regulatory pathways. Insulin/insulin-like (ILP) and target of rapamycin (TOR) nutrient signaling influence queen/worker caste development in honey bees [48], [49].

Two peptides with higher expression in worker-reared (putative gyne-destined) *P. metricus* larvae are homologous to an imaginal disc growth factor similar to proteins that have been previously described to cooperate with insulin to induce cellular proliferation, mobility, and polarization [74]. The shrub-like peptide is similar to shrub, which in *Drosophila* is important in development of the neuronal system [75], perhaps pointing to an early initiation of determination of differences that subsequently have been observed between brains of adult worker vs. gyne *P. metricus* [33], [34].

Our data suggest a role for lipid metabolism in *Polistes* caste development via up-regulation of mRNA for an apolipoprotein and a retinoid- and fatty acid-binding protein plus higher levels of apolipophorin-III protein. Lipid transport utilizes specialized lipoprotein complexes, the apolipoproteins, that stabilize the lipid components and mediate metabolism [76], thus further supporting the idea that nutrient allocation affects caste-biased larval development in *Polistes*. Enhanced lipid metabolism might lead to enhanced lipid depositories in gynes, helping them survive the temperate winter or tropical dry season [30], [77], [78]. Apolipoprotein was down-regulated in adult queens vs. workers of the tropical paper wasp *P. canadensis* [73].

Oxidoreductase is up-regulated in *A. mellifera* worker-destined larvae [46] and down-regulated in adult in-hive workers within nests vs. foragers that perform tasks in the field [45]. We found an oxidoreductase to be up-regulated in putative gyne-destined larvae. A serpin homologue is significantly up-regulated in cDNA libraries of late instar worker larvae and adult workers relative to those of queens in the yellowjacket *Vespula squamosa* [79]. We found higher serpin levels in putative gyne-destined larvae.

Our data suggest that several genes that are related to diapause in other insects are differentially expressed in foundress-reared and worker-reared larvae. This finding is consistent with the hypothesis that diapause physiology is foundational to the larval pathway divergence [21]. However, the finding is confounded by seasonal differences. Because foundress-reared and worker-reared larvae do not overlap in time, our data cannot explicitly rule out that the differences we observed between the larval groups reflect environmental patterning of seasonally varying temperature or day length that could affect adult phenotypes. This concern is diminished by the findings that pupae of *P. metricus* can diverge onto two developmental pathways under a single temperature and day length regime [37] and that our significant findings include phylogenetically conserved genes and proteins with pleiotropic phenotypic outcomes that include the adult stage in many animals as described above. Moreover, if larval gene transcript/protein level differences are regarded causal to adult phenotype, our data cannot distinguish between effects on expression that emerge from seasonally varying influences such as food quality or its availability in the environment relative to the hypothesized role of quantitatively different larval nourishment caused by solitary foundress vs. collective worker feeding behavior. Differences in food quality might be reflected in the finding that worker-destined and gyne-destined larvae of *P. metricus* differ significantly in nutrient levels [80]: gyne-destined larvae had higher levels of lipids and potassium, gained calories more quickly than worker-destined larvae, and stored more energy as they developed [80]. A literature search revealed no data in support of seasonal environmental differences in food quantity for *Polistes* nor suggestions that such differences might occur. In contrast, support for a role played by differences in the quantity of food fed to larvae can be drawn from studies in many species of *Polistes*, reviewed in [9], that document a pattern of change in size from small adults produced early in the season when only the foundress is present, to larger adults produced in mid-season when a worker force is present, with some diminishment in size near the end of the colony cycle [9]. Variation in larval feeding rates as a consequence of changes in the worker to larvae ratio [14], [82] and presence of gynes and males [9] have been hypothesized to be the basis for the seasonal patterning of body size. Direct support for the hypothesized effects of low versus high larval nourishment can be drawn from experiments in which food supplementation of *P. metricus* led to higher fat content in both the field [57] and lab [31], higher survival of a laboratory cold test [31], and, in the field, larger colony size, higher survivorship, and production of more gynes [56], [81]. Additionally, our data, in which most of the differentially-expressed genes and peptides/proteins are known to play roles in nutritionally-mediated physiological pathways, clearly mirror the regulatory importance of nutrition in caste-specific larval development of *A. mellifera* [82]. Larval determination of worker and queen castes in honey bees depends on a difference in food quality as well as food quantity [82]. In sum, there is considerable support for the hypothesis that difference in food quantity provisioned to larvae plays a major role in the larval divergence we have reported here.

Our findings from both gene and protein expression patterns favor rejection of the null hypothesis of no difference between foundress-reared and worker-reared larvae, and at the same time our findings fail to reject the hypothesis of larval pathway divergence presented by [21] and supported by [37]. This conclusion incorporates three major findings. Our first major finding that gene transcript and protein level data support a role for diapause regulatory factors during larval divergence in *Polistes metricus*. Our second major finding is that differentially expressed genes and proteins are active in physiological pathways that respond to differences in larval nutrition. Our third major finding is the differential expression in a primitively eusocial paper wasp of some of the same genes and peptides/proteins that are implicated in worker/queen developmental divergence in highly eusocial bees. Our data therefore suggest that caste regulatory mechanisms in advanced eusocial Hymenoptera with dimorphic worker and queen castes are foreshadowed by mechanisms affecting divergence onto two developmental pathways in monomorphic primitively eusocial Hymenoptera. We propose that larval pathway divergence, as exemplified by *Polistes*, may also have been the foundation for caste divergence in more derived eusocial forms.

In conclusion, our data add a new dimension of support for the larval divergence hypothesis. Quantification of differential gene expression and protein abundance between larvae that could correspond to the two developmental classes has heretofore been a missing link between a possible environmental trigger and a consequent response of larval divergence. Although not conclusive, our results indicate that differential larval nourishment may be the environmental trigger and that differential activity of the physiological machinery that regulates diapause-derived circuits may be the consequent response. Taken collectively, our results point the way to multiple avenues of genomic and proteomic inquiry into the regulation of the worker/gyne phenotype divergence, not only in *Polistes* paper wasps but also in other eusocial Hymenoptera. We believe that such research can contribute significantly to a broad and clear understanding of the evolutionary origin of caste in eusocial insects and its regulation by nutrition, hormones, genes, and proteins.

## Supporting Information

Table S1 Specimen identities, rearing conditions (FR  =  foundress-reared; WR  =  worker-reared); collection date, nest identities, and field site where collected. Field sites: Shaw  =  Shaw Nature Reserve near Gray Summit, Washington Co, Missouri; Tyson  =  Tyson Research Station near Eureka, St. Louis Co., Missouri. Specimen identities and nest numbers correspond to J. H. Hunt's field notes. The 2005 specimens were ancillary to a larger research project [1]. Nests were not used in the other study, and they were undisturbed after marking the foundress with a dot of paint until the dates of specimen collections. Each nest was collected en toto, rendering impossible the collection of FR and WR specimens from the same nest. Specimens for this study were restricted to large fifth-instar larvae that were of a size that soon would begin spinning their pupal cocoon. The middle letter of each specimen ID (e.g., C, E, H) indicates the colony of origin. The 2007 specimens were not part of a larger project, hence the different notation. Each FR specimen in 2007 came from a different nest. Several specimens were not included in the analyses for the reasons given. “Data outlier” indicates that the expression values for all genes were approximately 2X higher than for the other samples, thus the samples were clear outliers. Because specimens were numbered beginning with 1 for the largest larva and then in descending sequence by size, numbers 12 and 16 for the data outliers indicate that they were earlier in development than other larvae in the study. Exclusion of these samples decreased, rather than increased, significance values of statistical tests (data not shown). 1. Hunt JH, Kensinger BA, Kossuth J, Henshaw MT, Norberg K, et al. (2007) From casteless to castes - a diapause pathway underlies the gyne phenotype in Polistes paper wasps. Proceedings of the National Academy of Sciences USA 104: 14020-14025.
(0.06 MB DOC)

Table S2 Forward (FW) and reverse (RV) primer sequences for each putative gene used for quantitative real-time rtPCR of 5th instar larvae of Polistes metricus.
(0.06 MB DOC)

Table S3 Results of statistical comparisons of mRNA abundance for the 38 tested genes, comparing FR (foundress reared) and WR (worker reared) larvae. Genes significantly different between larval categories are in bold. “lm” indicates linear model ANOVA; “MWU” indicates Mann-Whitney U test.
(0.06 MB DOC)

Table S4 Microsatellite genotypes for worker reared (WR) *P. metricus* larvae. Larvae found to be heterozygous at one or more loci must be diploid, and thus female, while larvae that have only one allele at each locus are potentially male and were excluded from analyses.
(0.11 MB DOC)

Table S5 Peptides/proteins used in the MWU-test. Criteria for using a peptide/protein: spectrum count of at least 3 present for at least 3 out of 5 replicates. N = 5 samples per group. Note that the table contains redundant data. Peptides can match to different databases (Apis, Nasonia, Polistes contigs) and isoform sequences. Also, two different kinds of experiments were run. Several contigs can belong to the same protein. For some contigs peptides matched to more than one reading frame (can be distinguished by the Accession number). Red font  =  results from in-solution digestion. Black font  =  results from in-gel digestion. p-value  =  bootstrap analysis (1000 iterations) verified validity, i.e. probability of Type I error is not inflated by multiple comparisons.
(0.11 MB DOC)
