# Supplementary material for: Differential Gene Expression and Protein Abundance Evince Ontogenetic Bias toward Castes in a Primitively Eusocial Wasp
Source: PLoS One. 2010 May 17;5(5):e10674. doi: 10.1371/journal.pone.0010674 (PMC2871793; doi:10.1371/journal.pone.0010674)
Supplement: Table S1 — Specimen identities, rearing conditions (FR = foundress-reared; WR = worker-reared); collection date, nest identities, and field site where collected. Field sites: Shaw = Shaw Nature Reserve near Gray Summit, Washington Co, Missouri; Tyson = Tyson Research Station near Eureka, St. Louis Co., Missouri. Specimen identities and nest numbers correspond to J. H. Hunt's field notes. The 2005 specimens were ancillary to a larger research project [1]. Nests were not used in the other study, and they were undisturbed after marking the foundress with a dot of paint until the dates of specimen collections. Each nest was collected en toto, rendering impossible the collection of FR and WR specimens from the same nest. Specimens for this study were restricted to large fifth-instar larvae that were of a size that soon would begin spinning their pupal cocoon. The middle letter of each specimen ID (e.g., C, E, H) indicates the colony of origin. The 2007 specimens were not part of a larger project, hence the different notation. Each FR specimen in 2007 came from a different nest. Several specimens were not included in the analyses for the reasons given. “Data outlier” indicates that the expression values for all genes were approximately 2X higher than for the other samples, thus the samples were clear outliers. Because specimens were numbered beginning with 1 for the largest larva and then in descending sequence by size, numbers 12 and 16 for the data outliers indicate that they were earlier in development than other larvae in the study. Exclusion of these samples decreased, rather than increased, significance values of statistical tests (data not shown). 1. Hunt JH, Kensinger BA, Kossuth J, Henshaw MT, Norberg K, et al. (2007) From casteless to castes - a diapause pathway underlies the gyne phenotype in Polistes paper wasps. Proceedings of the National Academy of Sciences USA 104: 14020-14025. (0.06 MB DOC) [file pone.0010674.s001.doc]

Table S1.

| Genomics Specimens | | | | | |
| --- | --- | --- | --- | --- | --- |
| Specimen ID | Rearing Condition | Collection Date, 2005 | Nest # | Field Site | Excluded because |
| RC3 | FR | 8-Jun | 165 | Shaw |  |
| RC4 | FR | 8-Jun | 165 | Shaw |  |
| RE2 | FR | 14-Jun | 154 | Shaw |  |
| RE3 | FR | 14-Jun | 154 | Shaw |  |
| RE4 | FR | 14-Jun | 154 | Shaw |  |
| RE5 | FR | 14-Jun | 154 | Shaw |  |
| RH3 | FR | 19-Jun | 147 | Shaw |  |
| RH4 | FR | 19-Jun | 147 | Shaw |  |
| RH5 | FR | 19-Jun | 147 | Shaw |  |
| Ri1 | FR | 20-Jun | 4 | Tyson |  |
| Ri2 | FR | 20-Jun | 4 | Tyson | degraded RNA |
| Ri3 | FR | 20-Jun | 4 | Tyson | degraded RNA |
| RJ4 | WR | 30-Jul | 118 | Shaw |  |
| RJ5 | WR | 30-Jul | 118 | Shaw |  |
| RJ8 | WR | 30-Jul | 118 | Shaw |  |
| RK2 | WR | 30-Jul | 135 | Shaw |  |
| RP6 | WR | 3-Aug | 62 | Tyson |  |
| RP12 | WR | 3-Aug | 62 | Tyson | small larva, data outlier |
| RP16 | WR | 3-Aug | 62 | Tyson | small larva, data outlier |
| RQ4 | WR | 3-Aug | 53 | Tyson | degraded RNA |
| RQ6 | WR | 3-Aug | 53 | Tyson |  |
| RR4 | WR | 3-Aug | 53 | Tyson |  |
| Proteomics Specimens | | | | | |
| Specimen ID | Rearing Condition | Collection Date, 2007 | Nest # | Field Site | Excluded because |
| X-1 | FR | 18-Jun | X | Tyson |  |
| X-2 | FR | 18-Jun | X | Tyson |  |
| X-3 | FR | 18-Jun | X | Tyson |  |
| 17-2 | FR | 18-Jun | 17 | Tyson |  |
| 17-3 | FR | 18-Jun | 17 | Tyson | technical problems |
| 21-3 | FR | 18-Jun | 21 | Tyson |  |
| A1 | WR | 19-Jul | A | Tyson |  |
| A3 | WR | 19-Jul | A | Tyson |  |
| A4 | WR | 19-Jul | A | Tyson | technical problems |
| B1 | WR | 19-Jul | B | Tyson |  |
| C2 | WR | 19-Jul | C | Tyson |  |
| C8 | WR | 19-Jul | C | Tyson |  |
