# Supplementary material for: Differential Gene Expression and Protein Abundance Evince Ontogenetic Bias toward Castes in a Primitively Eusocial Wasp
Source: PLoS One. 2010 May 17;5(5):e10674. doi: 10.1371/journal.pone.0010674 (PMC2871793; doi:10.1371/journal.pone.0010674)
Supplement: Table S2 — Forward (FW) and reverse (RV) primer sequences for each putative gene used for quantitative real-time rtPCR of 5th instar larvae of Polistes metricus. (0.06 MB DOC) [file pone.0010674.s002.doc]

Table S2.

| *P. metricus* putative gene | FW primer | RV primer |
| --- | --- | --- |
| *PmHex70b (b)* | ATGGTTTGTGATTGAGACGATATTG | CCCTTAACAACGGTTTGGTAGTTG |
| *Pmusp* | GCCTGACAGAGGGTGATTAGGT | TCGCCAAGTGGACAGAACAA |
| *PmHex70b (a)* | AATAGATTATCCTCTTGTAGACCGATAAA | TGCTCTTGAACATTATTCAACATCCTTT |
| *PmPi3K* | CTCGCTCATTTATTAATTCGATCTCTCTA | TGCTAAGAAACATAGGAACGGACAA |
| *PmHsp8/HSC70* | CGGTGGGCTCGTTGATG | GCGTCAGGCAACCAAGGA |
| *PmHSP90alpha* | CCTTCCTGATAACTTTGAGAATCTTATTT | CAATTGCGAAGAAATTATTCCAGAA |
| *PmHsp90/Hsp90alpha* | CTCTTCTTCATCTTCTCCGACATCT | ATGATGAACCATCGAAGGAAATG |
| *PmTPX3* | TCCGTGCGCTTCGACAA | AAGCGTAAATGATTTGCCAGTTG |
| *PmHsp1alpha* | AAAGGTTCACCAGTGTCTCGTCTA | TGATGACGAACAATATTTATGGGAAT |
| *PmTPX1* | TCGTCGAGTACGCCATAATCG | GGTGGTATCGGTGAAATGAACA |
| *PmPCNA* | GGTTGTCCCATCTCTCAATG | CGCAACAATGTCCAAGATATTACG |
| *Pmoxidoreductase* | TTAGGAAAGATTATGGCCTTCTATGG | TGCACAGCCAACAGGAAATG |
| *PmRfaBp* | GTCCTCCATCAGTGACAGTTCCT | TACTACACGTACAGGCCTACTGTTCA |
| *PmSPARC* | GAGGTCACACCACTTCCAAATG | GCAACGCGAAGCTGAAACTAA |
| *PmRfaBp/APO* | AAATATCAAGCAACTTTGGACACAAGA | CGGCAGGTATGTTGAACTTCCT |
| *Pmtun* | CGTTGGCGCGTAGATCAGT | CGTGCAGGAAAGGACGAAGA |
| *PmILP2* | TCGTACATTGAATGAAATTGTCAATATT | CGCTCTCCAATTAGCAGCTGAT |
| *Pmg5sd* | CGACGTCGATGGTATTTACAATCT | CGAACTGAAGGTATGCAACATCTT |
| *Pmtif2B* | GCGATACGTTGATCATTTATGTGAGT | TGCAATACCTAGTGGCGATCAT |
| *PmIRS* | GTCTCTTTGAACGACTGCCTACAG | TCACGCAGCAGGAAGCAA |
| *PmTOR* | TCTTATTGGTTGGGTTCCACACT | TCTCTGATCAAAGTATGTAACGTATC |
| *PmCG9005-like* | CGCGAGGTCGAACGTCTT | GGAGGAAACTGATCGAGAAACAG |
| *Pmfax* | CAACTGCCCAAACCAAATGA | CCGAACAACGCAGCGTATCT |
| *PmeELF-1a* | CTCCAGTACCAGCAGCAACAAT | GCACCTGGACACAGAGATTTCA |
| *PmFAS* | GCCCATTGCGAACCCATA | ATTCGCGATGTCGGTCAATAT |
| *PmSh3Beta* | ACTTCCTTATTCCCGCTGATACC | GATAAGAAGTGGACACCGGCTAA |
| *PmCG11971-like* | CTTGACCAAACGACGCCAAT | GGCGGTAGGCCAGGATTT |
| *Pm chymotrypsin precursor* | TCCTGTTCCTTTGAAAGTTGTACCT | ATTTAACTCACTTGTCGGATTATGGA |
| *PmInos* | TCCCACTTGCCATTCAACATC | GATGATTGACTTCGCAAAGACATT |
| *PmEfl21* | GAATGATTACGATGCGATCAATG | CCTTTCTCGGTGCAGTTATGG |
| *PmTctp* | ACAGGCATGGATTCACCATCA | TCTATGGAGATCGACGGTTTAGTTG |
| *PmRPS8* | CCAGGCGCAGTGCTCTGTA | AAACTTGGACCTCAGCGTATTCA |
| *Pmmcp* | TGGCGCCAACACTTTGAA | TGCTGACCAATATGGAACTGATAAA |
| *PmInR2* | CCCAGATCATTATTGGGATACGTT | CCGCAAGCGTCTCTTCCA |
| *PmInR1* | CTGCCATCTTCAATATCAATTTCCT | CGAGTTTAGTCATATTGGATAATCCAAA |
| *PmAcCoASyn* | TTGCTAACCACAGCTGCTTCA | TTTGATGTCTACGGCCAGAAGGTAGAA |
| *PmCG5237-like* | ACCCATAACCGCCAACACCAT | TGCAGACATTGACGATGTTAGAAA |
| *PmClock* | TGACGACTTTATCCAAATCATCGA | GAGGTGCTAGGTACTTCCGGATAC |
| *Pmendopep* | TTCATTTCGCATTCGTTACGAT | TTTGTAGTGGAGAACGTCAATTCG |
| *PmVg1* | GGTAGCCCCATTGCTTCCA | TGCGCTTCAGTGATGTGGTAA |
