# Supplementary material for: Differential Gene Expression and Protein Abundance Evince Ontogenetic Bias toward Castes in a Primitively Eusocial Wasp
Source: PLoS One. 2010 May 17;5(5):e10674. doi: 10.1371/journal.pone.0010674 (PMC2871793; doi:10.1371/journal.pone.0010674)
Supplement: Table S3 — Results of statistical comparisons of mRNA abundance for the 38 tested genes, comparing FR (foundress reared) and WR (worker reared) larvae. Genes significantly different between larval categories are in bold. “lm” indicates linear model ANOVA; “MWU” indicates Mann-Whitney U test. (0.06 MB DOC) [file pone.0010674.s003.doc]

Table S3.

| *P. metricus* putative gene | Fval (lm) | p-value (lm) | MWU | p-val (MWU) |
| --- | --- | --- | --- | --- |
| ***PmPi3K*** | **18.889** | **0.001** | **4** | **0.0029** |
| ***PmAPO*** | **15.245** | **0.001** | **4** | **0.0029** |
| ***PmRfaBp*** | **11.008** | **0.005** | **6** | **0.0054** |
| ***Pmoxidoreductase*** | **15.178** | **0.001** | **7** | **0.0073** |
| ***PmHSC70*** | **10.025** | **0.006** | **8** | **0.0097** |
| ***PmSh3Beta*** | **7.177** | **0.017** | **10** | **0.0168** |
| ***PmSPARC*** | **9.066** | **0.009** | **11** | **0.0218** |
| ***PmIRS*** | **8.276** | **0.012** | **11** | **0.022** |
| ***PmeELF-1a*** | **4.997** | **0.041** | **12** | **0.028** |
| ***PmHex70b*** | **5.127** | **0.039** | **12** | **0.028** |
| ***PmILP2*** | **6.492** | **0.022** | **12** | **0.028** |
| ***PmTPX3*** | **6.681** | **0.021** | **12** | **0.0281** |
| ***Pmtif2B*** | **5.795** | **0.029** | **13** | **0.0359** |
| ***PmTPX1*** | **5.994** | **0.027** | **13** | **0.0359** |
| ***Pmusp*** | **6.770** | **0.02** | **13** | **0.0359** |
| ***PmHSP90alpha*** | **6.372** | **0.023** | **14** | **0.045** |
| *PmFAS* | 3.137 | 0.097 | 16 | 0.071 |
| *PmHSP90* | 4.071 | 0.062 | 18 | 0.107 |
| *Pmtun* | 2.748 | 0.118 | 18 | 0.107 |
| *Pmmcp* | 3.590 | 0.078 | 18 | 0.107 |
| *Pmg5sd* | 2.596 | 0.128 | 20 | 0.157 |
| *PmCG11971-like* | 2.450 | 0.14 | 18 | 0.169 |
| *PmCG9005-like* | 2.018 | 0.176 | 21 | 0.188 |
| *Pmfax* | 1.598 | 0.225 | 21 | 0.188 |
| *PmVg* | 2.313 | 0.149 | 21 | 0.188 |
| *PmClock* | 2.040 | 0.174 | 24 | 0.306 |
| *PmHSP1alpha* | 2.443 | 0.139 | 25 | 0.354 |
| *PmTOR* | 2.685 | 0.122 | 25 | 0.354 |
| *PmInos* | 0.614 | 0.446 | 26 | 0.407 |
| *PmPCNA* | 0.457 | 0.509 | 27 | 0.464 |
| *Pmchymotrypsin* | 1.235 | 0.284 | 28 | 0.523 |
| *PmCG5237-like* | 0.639 | 0.437 | 29 | 0.591 |
| *PmEfl21* | 0.075 | 0.788 | 30 | 0.661 |
| *PmInR2* | 0.045 | 0.835 | 31 | 0.733 |
| *PmInR1* | 0.038 | 0.849 | 27 | 0.786 |
| *Pmendopeptidase* | 0.003 | 0.957 | 32 | 0.807 |
| *PmACAS* | 0.030 | 0.865 | 33 | 0.884 |
| *Pmtctp* | 0.126 | 0.728 | 34 | 0.961 |
