# Supplementary material for: Differential Gene Expression and Protein Abundance Evince Ontogenetic Bias toward Castes in a Primitively Eusocial Wasp
Source: PLoS One. 2010 May 17;5(5):e10674. doi: 10.1371/journal.pone.0010674 (PMC2871793; doi:10.1371/journal.pone.0010674)
Supplement: Table S4 — Microsatellite genotypes for worker reared (WR) P. metricus larvae. Larvae found to be heterozygous at one or more loci must be diploid, and thus female, while larvae that have only one allele at each locus are potentially male and were excluded from analyses. (0.11 MB DOC) [file pone.0010674.s004.doc]

Table S4a: 2005 Specimens:

| Specimen ID | Microsatellite Locus | | | | | | Sex | # Heterozy-gous Loci | # Non-amplifying loci |
| --- | --- | --- | --- | --- | --- | --- | --- | --- | --- |
|  | Pbe128 | Pbe269 | Pbe203 | Pbe205 | Pbe424 | Pbe440 |  |  |  |
| RC2 |  |  |  |  |  |  | dud |  | 6 |
| RC3 | 191/194 | 158/164 | 153/159 | 250/253 | 244/253 | 235/247 | F | 6 |  |
| RC4 | 191/191 | 161/164 | 153/159 | 250/253 | 244/253 | 235/247 | F | 5 |  |
| RE2 | 191/194 | 158/161 | 153/171 | 253/259 | 235/250 | 244/244 | F | 5 |  |
| RE3 | 188/191 | 158/161 | 153/171 | 250/259 | 250/250 | 235/244 | F | 5 |  |
| RE4 | 191/194 | 158/161 | 171/177 | 250/259 | 250/250 | 235/244 | F | 5 |  |
| RE5 | 191/194 | 158/158 | 153/171 | 250/259 | 250/250 | 244/244 | F | 3 |  |
| RH3 | 197/200 | 161/164 | 171/174 | 256/262 | 250/250 | 238/247 | F | 5 |  |
| RH4 | 197/200 | 164/170 | 171/174 | 256/256 | 247/250 | 238/247 | F | 5 |  |
| RH5 | 197/200 | 164/170 | 174/190 | 256/256 | 247/250 | 238/247 | F | 5 |  |
| Ri1 | 188/197 | 161/161 | 168/174 | 250/256 | 238/244 | 238/241 | F | 5 |  |
| Ri2 | 188/188 | 161/164 | 153/168 | 250/256 | 238/244 | 241/256 | F | 5 |  |
| Ri3 | 188/197 | 161/164 | 168/174 | 250/256 | 238/244 | 241/256 | F | 6 |  |
| RJ4 | 188/197 | 164/173 | 171/171 | 250/250 | 244/250 | 235/253 | F | 4 |  |
| RJ5 | 191/191 | 161/167 | 165/174 | 250/256 | 247/250 | 247/268 | F | 5 |  |
| RJ6 | 191 | 173 | 171 | 250 | 244 | 235 | M | 0 |  |
| RJ7 | 191 | 167 | 165 |  | 250 | 235 | M | 0 | 1 |
| RJ8 | 191/191 | 161/167 |  | 250/256 | 247/250 | 247/268 | F | 4 | 1 |
| RJ9 | 191 | 173 | 165 | 250 | 250 | 235 | M | 0 |  |
| RK2 | 194/200 | 164/164 | 171/171 | 250/250 | 238/244 | 235/259 | F | 3 |  |
| RL4 | 191 | 158 | 181 |  | 244 | 256 | M | 0 | 1 |
| RL5 | 188 | 164 | 180 | 250 | 250 | 238 | M | 0 |  |
| RL6 | 191/191/191 | 158/161/164 | 153/162/168 | 250/250/250 | 244/262 | 235/247/256 | triploid |  |  |
| RL7 | 191 | 158 | 162 | 250 | 250 | 256 | M | 0 |  |
| RL8 | 191 | 164 | 162 | 250 | 244 | 256 | M | 0 |  |
| RO5 | 188 | 161 | 168 | 254 | 232 | 244 | M | 0 |  |
| RO6 | 188 | 161 | 168 | 250 | 247 | 256 | M | 0 |  |
| RO7 | 188 | 161 | 204 | 250 | 247 | 244 | M | 0 |  |
| RO10 | 188 | 161 | 168 | 250 | 247 | 256 | M | 0 |  |
| RO12 | 188 | 158 | 204 | 254 | 232 | 244 | M | 0 |  |
| RO14 | 188 | 161 | 204 | 250 | 247 | 244 | M | 0 |  |
| RP6 | 203/203 | 164/164 | 171/177 | 250/254 | 244/247 | 253/265 | F | 4 |  |
| RP8 | 203 | 161 | 168 | 254 | 247 | 244 | M | 0 |  |
| RP11 | 191 | 164 | 168 | 254 | 244 | 253 | M | 0 |  |
| RP12 | 191/203 | 164/164 | 168/177 | 250/254 | 244/247 | 244/265 | F | 5 |  |
| RP16 | 203/203 | 164/164 | 171/177 | 250/254 | 247/247 | 253/265 | F | 3 |  |
| RQ4 | 194/200 | 164/173 | 159/159 | 250/254 | 241/259 |  | F | 4 | 1 |
| RQ5 | 194 | 173 | 159 | 250 | 250 |  | M | 0 | 1 |
| RQ6 | 194/200 | 164/164 | 153/159 | 250/250 | 241/250 |  | F | 3 | 1 |
| RR4 | 191/194 | 155/164 | 171/174 | 250/257 | 244/244 | 235/238 | F | 5 |  |
| RR5 | 194 | 164 | 168 | 250 | 244 | 235 | M | 0 |  |
| RR6 | 194 | 164 | 174 | 259 | 253 | 238 | M | 0 |  |

Table S4b: 2007 Specimens:

| Specimen ID | Microsatellite Locus | | | | | | Sex | # Heterozy-gous Loci | # Non-amplifying loci |
| --- | --- | --- | --- | --- | --- | --- | --- | --- | --- |
|  | Pbe128 | Pbe203 | Pbe205 | Pbe269 | Pbe424 | Pbe440 |  |  |  |
| A1 | 191/197 | 156/165 | 252/255 | 161/173 |  |  | F | 4 | 2 |
| A2 | 191 | 165 | 252 | 173 | 245 | 236 | M | 0 |  |
| A3 | 191/197 | 165/165 | 255/258 | 161/173 |  |  | F | 3 | 2 |
| A4 | 191/197 | 156/165 | 252/255 | 161/161 | 245/254 |  | F | 4 | 1 |
| A5 | 191 | 156 | 252 | 161 | 245 | 251 | M | 0 |  |
| A6 | 188 | 165 | 258 | 161 |  |  | M | 0 | 2 |
| A7 | 191 | 156 | 258 | 173 |  |  | M | 0 | 2 |
| A8 | 191 | 156 | 252 | 173 | 245 | 251 | M | 0 |  |
| A9 | 191 | 156 | 252 | 173 | 245 |  | M | 0 | 1 |
| B1 | 191/191 | 168/183 | 252/258 | 161/167 |  | 242/245 | F | 4 | 1 |
| B2 | 191 | 165 | 252 | 167 | 248 | 266 | M | 0 |  |
| B3 | 191 | 183 | 252 | 161 |  | 242 | M | 0 | 1 |
| B4 | 191 | 165 | 252 | 161 |  |  | M | 0 | 2 |
| C1 | 194 | 180 | 252 | 161 | 248 | 245 | M | 0 |  |
| C2 | 188/194 | 159/180 | 252/252 | 161/170 | 245/248 | 239/245 | F | 5 |  |
| C3 | 188 | 195 | 252 | 161 | 248 |  | M | 0 | 1 |
| C4 | 188 | 195 | 252 | 170 | 245 |  | M | 0 | 1 |
| C5 | 188 | 195 | 252 | 161 |  |  | M | 0 | 2 |
| C6 | 188 | 180 | 252 | 161 | 245 | 248 | M | 0 |  |
| C7 | 188 | 180 | 252 | 161 | 245 | 245 | M | 0 |  |
| C8 |  | 159/180 |  | 161/161 |  | 239/248 | F | 2 | 3 |
| C9 | 188 | 195 | 252 | 161 | 248 | 248 | M | 0 |  |
| C10 | 188 | 180 | 252 | 161 |  | 245 | M | 0 | 1 |
