# Supplementary material for: Differential Gene Expression and Protein Abundance Evince Ontogenetic Bias toward Castes in a Primitively Eusocial Wasp
Source: PLoS One. 2010 May 17;5(5):e10674. doi: 10.1371/journal.pone.0010674 (PMC2871793; doi:10.1371/journal.pone.0010674)
Supplement: Table S5 — Peptides/proteins used in the MWU-test. Criteria for using a peptide/protein: spectrum count of at least 3 present for at least 3 out of 5 replicates. N = 5 samples per group. Note that the table contains redundant data. Peptides can match to different databases (Apis, Nasonia, Polistes contigs) and isoform sequences. Also, two different kinds of experiments were run. Several contigs can belong to the same protein. For some contigs peptides matched to more than one reading frame (can be distinguished by the Accession number). Red font = results from in-solution digestion. Black font = results from in-gel digestion. p-value = bootstrap analysis (1000 iterations) verified validity, i.e. probability of Type I error is not inflated by multiple comparisons. (0.11 MB DOC) [file pone.0010674.s005.doc]

Table S5.

| Accession nr. | Contig/similar to | p-value |
| --- | --- | --- |
| **Represented in the figures** | | |
| **AAN63347** | **AgSP-1 arylphorin [Anthonomus grandis]** | **0.008** |
| **28945_dbpolistesnewandrest.fasta** | **(Contig)32130** | **0.016** |
| **AAN63347** | **AgSP-1 arylphorin [Anthonomus grandis]** | **0.016** |
| **18257_dbpolistesnewandrest.fasta** | **(Contig)46732** | **0.016** |
| **17531_dbpolistesnewandrest.fasta** | **(Contig)46611** | **0.032** |
| **XP_393451** | **PREDICTED: similar to Heterogeneous nuclear ribonucleoprotein at 27C CG10377-PA, isoform A [Apis mellifera]** | **0.032** |
| **18256_dbpolistesnewandrest.fasta** | **(Contig)46732** | **0.032** |
| **18480_dbpolistesnewandrest.fasta** | **(Contig)46769** | **0.056** |
| **15221_dbpolistesnewandrest.fasta** | **(Contig)40765** | **0.056** |
| **18185_dbpolistesnewandrest.fasta** | **(Contig)46720** | **0.056** |
| Not represented in the figures |  |  |
| 10212_dbpolistesnewandrest.fasta | (Contig)45391 | 0.095 |
| 18478_dbpolistesnewandrest.fasta | (Contig)46769 | 0.095 |
| XP_001605116 | PREDICTED: similar to cytochrome P450 [Nasonia vitripennis] | 0.095 |
| 25158_dbpolistesnewandrest.fasta | (Contig)9654 | 0.095 |
| 13063_dbpolistesnewandrest.fasta | (Contig)24022 | 0.095 |
| XP_001122907 | PREDICTED: similar to Ccp84Ad CG2341-PA [Apis mellifera] | 0.151 |
| 18257_dbpolistesnewandrest.fasta | (Contig)46732 | 0.151 |
| 12538_dbpolistesnewandrest.fasta | (Contig)45779 | 0.151 |
| 6761_dbpolistesnewandrest.fasta | (Contig)44816 | 0.151 |
| XP_001608067 | PREDICTED: hypothetical protein [Nasonia vitripennis] | 0.222 |
| 10518_dbpolistesnewandrest.fasta | (Contig)34520 | 0.222 |
| XP_001607209 | PREDICTED: similar to ENSANGP00000019367 [Nasonia vitripennis] | 0.222 |
| 8170_dbpolistesnewandrest.fasta | (Contig)17745 | 0.222 |
| 11824_dbpolistesnewandrest.fasta | (Contig)45660 | 0.222 |
| XP_392104 | PREDICTED: similar to CG31075-PA isoform 1 [Apis mellifera] | 0.222 |
| XP_623252 | PREDICTED: similar to CG31075-PA [Apis mellifera] | 0.222 |
| 15221_dbpolistesnewandrest.fasta | (Contig)40765 | 0.222 |
| 18256_dbpolistesnewandrest.fasta | (Contig)46732 | 0.310 |
| XP_625004 | PREDICTED: similar to CG15006-PA [Apis mellifera] | 0.310 |
| 9155_dbpolistesnewandrest.fasta | (Contig)45215 | 0.310 |
| 17832_dbpolistesnewandrest.fasta | (Contig)46661 | 0.310 |
| 6761_dbpolistesnewandrest.fasta | (Contig)44816 | 0.310 |
| XP_624156 | PREDICTED: similar to ATP synthase- CG11154-PA, isoform A [Apis mellifera] | 0.310 |
| 8302_dbpolistesnewandrest.fasta | (Contig)6845 | 0.310 |
| 18766_dbpolistesnewandrest.fasta | (Contig)46817 | 0.310 |
| 18160_dbpolistesnewandrest.fasta | (Contig)46716 | 0.310 |
| 13612_dbpolistesnewandrest.fasta | (Contig)45958 | 0.310 |
| 11068_dbpolistesnewandrest.fasta | (Contig)23689 | 0.310 |
| 3235_dbpolistesnewandrest.fasta | (Contig)38767 | 0.421 |
| 17249_dbpolistesnewandrest.fasta | (Contig)46564 | 0.421 |
| XP_001600045 | PREDICTED: similar to ENSANGP00000014839 [Nasonia vitripennis] | 0.421 |
| 18713_dbpolistesnewandrest.fasta | (Contig)41347 | 0.421 |
| 16783_dbpolistesnewandrest.fasta | (Contig)30103 | 0.421 |
| XP_625004 | PREDICTED: similar to CG15006-PA [Apis mellifera] | 0.421 |
| XP_623046 | PREDICTED: similar to Tropomyosin 1 CG4898-PD, isoform D [Apis mellifera] | 0.548 |
| XP_623393 | PREDICTED: similar to Tropomyosin 1 CG4898-PL, isoform L [Apis mellifera] | 0.548 |
| XP_623470 | PREDICTED: similar to Tropomyosin 1 CG4898-PB, isoform B isoform 2 [Apis mellifera] | 0.548 |
| 18258_dbpolistesnewandrest.fasta | (Contig)46732 | 0.548 |
| 622_dbpolistesnewandrest.fasta | (Contig)43793 | 0.548 |
| 30469_dbpolistesnewandrest.fasta | (Contig)43306 | 0.548 |
| 17970_dbpolistesnewandrest.fasta | (Contig)46684 | 0.548 |
| XP_001605359 | PREDICTED: similar to protein disulfide isomerase [Nasonia vitripennis] | 0.548 |
| 18479_dbpolistesnewandrest.fasta | (Contig)46769 | 0.548 |
| 17789_dbpolistesnewandrest.fasta | (Contig)46654 | 0.548 |
| 4241_dbpolistesnewandrest.fasta | (Contig)38935 | 0.548 |
| 18768_dbpolistesnewandrest.fasta | (Contig)46817 | 0.548 |
| XP_001122907 | PREDICTED: similar to Ccp84Ad CG2341-PA [Apis mellifera] | 0.548 |
| XP_394187 | PREDICTED: similar to Nucleoplasmin CG7917-PA isoform 1 [Apis mellifera] | 0.548 |
| 4404_dbpolistesnewandrest.fasta | (Contig)44423 | 0.690 |
| 6136_dbpolistesnewandrest.fasta | (Contig)44712 | 0.690 |
| XP_623282 | PREDICTED: similar to ERp60 CG8983-PA, isoform A isoform 2 [Apis mellifera] | 0.690 |
| 18767_dbpolistesnewandrest.fasta | (Contig)46817 | 0.690 |
| 23879_dbpolistesnewandrest.fasta | (Contig)31286 | 0.690 |
| 7723_dbpolistesnewandrest.fasta | (Contig)39515 | 0.690 |
| XP_392125 | PREDICTED: similar to Tropomyosin 1 CG4898-PD, isoform D isoform 1 [Apis mellifera] | 0.841 |
| 9667_dbpolistesnewandrest.fasta | (Contig)34378 | 0.841 |
| 18185_dbpolistesnewandrest.fasta | (Contig)46720 | 0.841 |
| NP_001011603 | arginine kinase [Apis mellifera] | 0.841 |
| XP_001607122 | PREDICTED: similar to arginine kinase-like protein [Nasonia vitripennis] | 0.841 |
| 3107_dbpolistesnewandrest.fasta | (Contig)44207 | 0.841 |
| XP_001606463 | PREDICTED: similar to ENSANGP00000012893 [Nasonia vitripennis] | 0.841 |
| 18184_dbpolistesnewandrest.fasta | (Contig)46720 | 0.841 |
| 1228_dbpolistesnewandrest.fasta | (Contig)43894 | 0.841 |
| XP_392899 | PREDICTED: similar to 60 kDa heat shock protein, mitochondrial precursor (Hsp60) (60 kDa chaperonin) (CPN60) (Heat shock protein 60) (HSP-60) (Mitochondrial matrix protein P1) [Apis mellifera] | 0.841 |
| 8045_dbpolistesnewandrest.fasta | (Contig)45030 | 0.841 |
| NP_001011572 | transferrin [Apis mellifera] | 0.841 |
| XP_001608067 | PREDICTED: hypothetical protein [Nasonia vitripennis] | 0.841 |
| XP_001120364 | PREDICTED: similar to 60S acidic ribosomal protein P2 (Acidic ribosomal protein RPA1) [Apis mellifera] | 0.841 |
| 1174_dbpolistesnewandrest.fasta | (Contig)43885 | 0.841 |
| XP_001599003 | PREDICTED: similar to tropomyosin 1 [Nasonia vitripennis] | 1.000 |
| XP_391961 | PREDICTED: similar to Tropomyosin 2 CG4843-PB, isoform B [Apis mellifera] | 1.000 |
| 6888_dbpolistesnewandrest.fasta | (Contig)44837 | 1.000 |
| 12312_dbpolistesnewandrest.fasta | (Contig)45741 | 1.000 |
| XP_001600126 | PREDICTED: similar to CG4898-PB [Nasonia vitripennis] | 1.000 |
| 25775_dbpolistesnewandrest.fasta | (Contig)42524 | 1.000 |
| 9047_dbpolistesnewandrest.fasta | (Contig)45197 | 1.000 |
| 9430_dbpolistesnewandrest.fasta | (Contig)45261 | 1.000 |
| XP_624357 | PREDICTED: similar to supercoiling factor CG9148-PA, isoform A [Apis mellifera] | 1.000 |
| XP_393090 | PREDICTED: similar to Heat shock protein cognate 3 CG4147-PA, isoform A [Apis mellifera] | 1.000 |
| 14212_dbpolistesnewandrest.fasta | (Contig)46058 | 1.000 |
| 14153_dbpolistesnewandrest.fasta | (Contig)46048 | 1.000 |
| 6794_dbpolistesnewandrest.fasta | (Contig)33899 | 1.000 |
| 3016_dbpolistesnewandrest.fasta | (Contig)44192 | 1.000 |
| 18688_dbpolistesnewandrest.fasta | (Contig)46804 | 1.000 |
| XP_394765 | PREDICTED: similar to CG32687-PA [Apis mellifera] | 1.000 |
| XP_396769 | PREDICTED: similar to Imaginal disc growth factor 4 CG1780-PA, isoform A [Apis mellifera] | 1.000 |
| XP_001120887 | PREDICTED: similar to Imaginal disc growth factor 4 CG1780-PA, isoform A, partial [Apis mellifera] | 1.000 |
| 18689_dbpolistesnewandrest.fasta | (Contig)46804 | 1.000 |
| 14384_dbpolistesnewandrest.fasta | (Contig)35164 | 1.000 |
| XP_001604765 | PREDICTED: similar to ENSANGP00000015354 [Nasonia vitripennis] | 1.000 |
| XP_391915 | PREDICTED: similar to CG32209-PB [Apis mellifera] | 1.000 |
| 18184_dbpolistesnewandrest.fasta | (Contig)46720 | 1.000 |
| 16950_dbpolistesnewandrest.fasta | (Contig)46514 | 1.000 |
